# Integration and Co-design of Memristive Devices and Algorithms for Artificial Intelligence

**DOI:** 10.1016/j.isci.2020.101809

**Published:** 2020-11-17

**Authors:** Wei Wang, Wenhao Song, Peng Yao, Yang Li, Joseph Van Nostrand, Qinru Qiu, Daniele Ielmini, J. Joshua Yang

**Affiliations:** 1 Dipartimento di Elettronica, Informazione e Bioingegneria, Politecnico di Milano and IU.NET, Piazza L. da Vinci 32, Milano 20133, Italy; 2 Electrical and Computer Engineering Department, University of Southern California, Los Angeles, CA, USA; 3 The Andrew and Erna Viterbi Department of Electrical Engineering, Technion-Israel Institute of Technology, Haifa 32000, Israel; 4 Air Force Research Laboratory, Information Directorate, Rome, NY, USA; 5 Electrical Engineering and Computer Science Department, Syracuse University, NY, USA

**Keywords:** Computer Architecture, Hardware Co-design, Materials Science

## Abstract

Memristive devices share remarkable similarities to biological synapses, dendrites, and neurons at both the physical mechanism level and unit functionality level, making the memristive approach to neuromorphic computing a promising technology for future artificial intelligence. However, these similarities do not directly transfer to the success of efficient computation without device and algorithm co-designs and optimizations. Contemporary deep learning algorithms demand the memristive artificial synapses to ideally possess analog weighting and linear weight-update behavior, requiring substantial device-level and circuit-level optimization. Such co-design and optimization have been the main focus of memristive neuromorphic engineering, which often abandons the “non-ideal” behaviors of memristive devices, although many of them resemble what have been observed in biological components. Novel brain-inspired algorithms are being proposed to utilize such behaviors as unique features to further enhance the efficiency and intelligence of neuromorphic computing, which calls for collaborations among electrical engineers, computing scientists, and neuroscientists.

## Graphical Abstract

**Figure:**
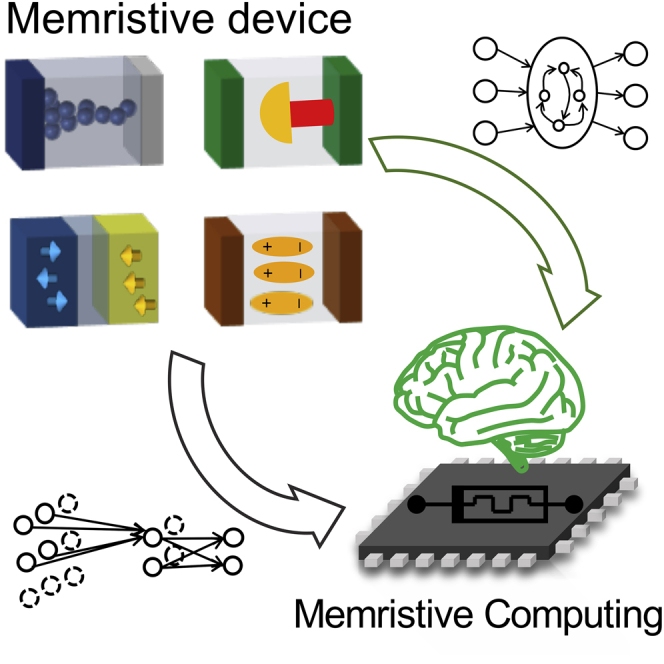

## Introduction

**Figure 1:**
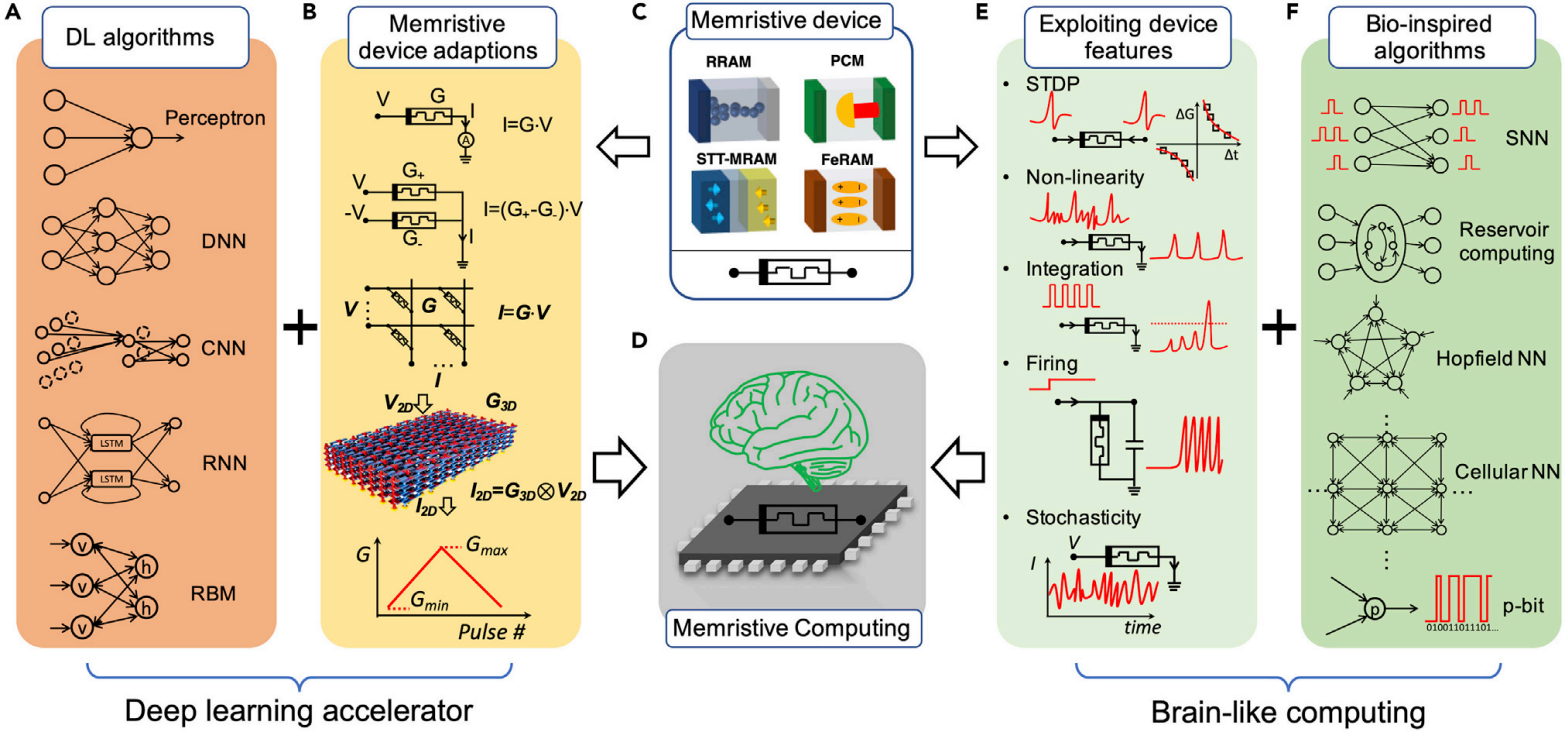
Integration of Learning Algorithms and Memristive Devices for Memristive Neuromorphic Computing (A) Various algorithms of deep learning (DL) neural networks, including simple perceptron, deep (multiplayer) neural network (DNN), convolutional neural network (CNN), recurrent neural network (RNN), and restricted Boltzmann machine (RBM). (B) Adaptions and performance enhancements of memristive devices for their synaptic application in DL algorithms: linear read of the memristive device, realization of both positive and negative weight via differential pair, mapping vector matrix multiplication and accumulation (VMMA) to 2D and 3D memristive array, and linear conductance update for identical pulses. Reproduced from (Lin et al., 2020), copyright © 2020, Springer Nature. (C) Various memristive devices, including resistive random-access memory (RRAM), phase-change memory (PCM), spin-torque transfer random access memory (STT-RAM), and ferroelectric random access memory (FeRAM). (D) Illustration of a memristive neuromorphic computing system integrated within a monolithic chip. (E) Unique memristive device features for emerging algorithms, such as the capability of spike-timing dependent plasticity (STDP), nonlinearity for filtering dendrites, integration and firing functions for memristive neurons, and stochasticity. (F) Emerging algorithms and architectures can be implemented by memristive devices, including spiking neural network (SNN), reservoir computing, Hopfield neural network (Hopfield NN), cellular neural network (Cellular NN), and a probabilistic bit (p-bit).

Artificial intelligence (AI) has made great progress in recent years with the help of the advances of deep learning (DL) technologies (LeCun et al. 2015). However, owing to the high volume of data needed to be frequently transferred between processing units and memories, the performance of deep learning algorithms is limited by the von Neumann bottleneck in conventional computers. The existing von Neumann bottleneck can be overcome by in-memory computing with memristive devices, where the computation takes place in the analog domain in the memory, right at the data location. Memristive devices, existing in several forms, such as resistive switching random access memory (RRAM), phase-change memory (PCM), magnetic random access memory (MRAM), and ferroelectric random access memory (FeRAM) (Figure 1C), have tunable conductance states, similar to the plasticity of biological synapses (Ielmini and Wong, 2018; Wang et al., 2020c), and thus can enable in-memory computing, in analogy to the biological neural system. Thanks to their scalability, stacking-ability, simple device structure, and other intriguing properties, the memristive devices have been considered as leading candidates for synaptic devices for hardware implementation of neural networks and machine learning, providing an energy-efficient and low-latency solution for future AI (Yang et al., 2013; Ielmini and Wong, 2018).

With their ionic transport mechanisms similar to the molecular activities in biological intelligent systems, the memristive devices also exhibit rich dynamics, resembling synaptic and neuronal dynamics found in biological systems. For instance, stochastic switching (Mizrahi et al., 2018), pulse-pair facilitation (Wu et al., 2018), and short-term plasticity (Ohno et al., 2011) have been observed in memristors and are suitable to reproduce the dynamics of biological synapses. This may eventually lead to computational systems capable of faithfully emulating the information representation and processing in the brain with much-improved energy efficiency and fidelity over the conventional systems.

Previous efforts to implement these algorithms that take advantage of memristive devices have met with limited success for two reasons: (1) Current state-of-the-art DL technologies use digitalized values (floating-point in software solutions or integers in CMOS-based hardware accelerators) for connection weights. Representing these weights using memristive conductance has suffered from non-ideal behaviors. (2) There are currently no complete algorithms to exploit the bio-plausible behaviors of the memristive devices. As a result, there exist substantial mismatches between algorithms and device properties, and therefore the integration of learning algorithms and memristive devices via co-design is imperative. Specifically, mismatches between memristive devices and learning algorithms can be addressed not only from the device side to engineer the materials for “expected” properties, which has been pursued intensively so far, but also from the algorithm side to either compensate the nonidealities of the devices or exploit some of the “unexpected” properties as valuable features for new types of computing, which has been relatively less explored.

In this perspective, recent achievements in the co-design of memristive devices and learning algorithms are reviewed, to provide a comprehensive overview of the status and remaining challenges for future explorations. We first give an overview of the co-design efforts of learning algorithms and memristive devices (section “The Integration of Learning Algorithms and Memristive Devices”). We then review such co-design efforts in depth from three standpoints: (1) compensating the device nonidealities at the single synapse level and memristive array level in vector-matrix multiplication and accumulation (VMMA) (section “DL Accelerators by Memristive Hardware “); (2) exploiting unique memristive device features in various bio-inspired learning algorithms (section “Exploiting Memristive Properties for Brain-Inspired Algorithms”); and (3) constructing brain-like computing systems with the bio-inspired algorithms enabled by unique memristive features (section “Toward a Brain-like Computing System”).

### The Integration of Learning Algorithms and Memristive Devices

The artificial neural network (ANN) has a lot of variants, such as the simple perceptron, deep neural network (DNN), deep convolutional neural network (CNN), recurrent neural network (RNN), and deep belief neural network based on restricted Boltzmann machines (RBMs) (Figure 1A), all belonging to the large family of DL techniques (LeCun et al., 2015). The operation of the ANN can be divided into two stages, inference and learning. During the inference phase, each layer of the neural network transforms the input signals by multiplication with the synaptic weights, summation at each output neuron, and activation according to a non-linear function. During the learning phase, the network is trained with data to adjust the synaptic weights for correct inference. Most of the learning tasks can be technically divided into three categories, supervised learning, unsupervised learning, and reinforcement learning, depending on the type and availability of feedback. In most approaches, an objective function or loss function is defined for weight training, that is, how good (or bad) the current weight configuration is, to fulfill the application (e.g., classification or decision) requirement, and the goal of training is to minimize the loss function or maximize the reward.

The most successful learning rule so far is error backpropagation (BP), where the loss function (or error) in the last layer is back-propagated to the preceding layers via the synaptic networks (Rumelhart et al., 1986). BP solves the credit (or blame) assignment problem, i.e., the weight updates to decrease the error, by calculating the gradients of the objective function with respect to the network parameters.

In the DL networks of Figure 1A, the massive synaptic connections and matrix-vector multiplications can be implemented physically within the memristive devices as illustrated in Figure 1B. Memristive devices perform the in-memory computation of the synaptic weighting functions in both forward inference and backward error propagation. Thanks to their ability to nanoscale miniaturization (Figure 1C), hardware acceleration with high efficiency for DL techniques can be achieved (Figure 1D). However, several technical issues still need to be addressed to commercialize such memristive DL accelerators at large scales.

Spiking neural networks (SNNs) take inspirations from neuroscience (Maass 1997) (Figure 1F), by preserving a more biological behavior of the spiking neuron, which can emit spikes in response to a spiking input stimulation. Spike-timing dependent plasticity (STDP) (Bi and Poo 1998) is a widely discussed synaptic property that has been realized by memristive devices and extensively reported (Figure 1E). A major limitation of the local learning rules such as STDP is that they can only be applied to shallow networks with only one layer. Recent research works aim at pushing the network deeper by introducing backpropagation approximation in spike domain (Shrestha et al., 2019), using synthetic local gradient (Kaiser et al., 2020) and adopting backward residual connections and stochastic SoftMax functions (Panda et al., 2020).

Artificial spiking neurons can mimic closely the biological neurons' behaviors, for example, reproducing the exact waveform of the membrane potential in different phases of a fire. However, these bio-realistic models are rarely used in software-simulated SNNs owing to a heavier computational burden. More typically, spiking neurons in SNNs are designed to have a binary response, i.e., “1” when the neuron receives or generates a spike or “0” when it does not. Memristive devices with non-linearity, state-variable accumulation (integration), volatility, as well as stochasticity (Figure 1E) can be used to construct spiking neurons and dendrites, thus combining bio-realistic responses with low computational burdens via the physical properties of the memristor.

There are various methods to encode the information into the spikes, such as using the frequency of a train of spikes (rate coding) or the precise arriving time of each spike (temporal coding) (Masquelier et al., 2009). Different encoding methods running on different neural networks require different training rules, which, however, are usually more computationally expensive than ANNs. Temporal-encoded SNNs are more efficient than rate-encoded SNNs, as the information can be contained in just one spike. Owing to the time-related nature, temporal-coded SNNs are more suitable to process time-related data, such as speech, sound, and vision. Memristive devices with state-variable accumulation, or short-term volatility, (Figure 1E) can be used to process these temporal-encoded spiking patterns in reservoir computing networks (Moon et al., 2019) (Figure 1F). Other advanced methods have been proposed to employ a pseudo-gradient to overcome the non-differentiability of a spiking neuron in feedforward networks. For instance, the e-prop training method in recurrent SNN (Bellec et al., 2020) has been proposed to approximate the backpropagation through time (BPTT) algorithm for traditional RNNs (Werbos 1988), which removes biologically unrealistic computational requirements and makes it possible to build on-chip hardware learning units.

Besides the SNNs as a signal morphological approach, another approach for brain-like computation is the collective-state computation that emulates brain-like computation at a high level (Csaba and Porod 2020). This computational model originated from the Hopfield neural network (Hopfield 1984), then extended to cellular neural networks (Chua and Yang 1988), coupled oscillators (Ignatov et al., 2017), and adiabatic annealing machines with probabilistic bits (Borders et al., 2019) (Figure 1F). In these networks, nonlinear synaptic and neural behaviors as well as probabilistic behaviors are needed, which can be provided by the memristive devices (Figure 1E). Similarities between memristive devices and biological components, such as synapses, dendrites, and neurons, have been extensively reported. Some fundamental functionalities or brain-inspired algorithms have also been demonstrated. However, practical computing systems based on these algorithms have rarely been reported.

### DL Accelerators by Memristive Hardware

Memristive synapses can be optimized to accelerate DL algorithms. Assembled into a crossbar array configuration, memristive devices are inherently suitable for efficient VMMA operations, which account for a majority percentage of the computation in artificial neural networks, by directly using Ohm's law for multiplications and Kirchhoff's current law for accumulations. Both passive (Prezioso et al., 2015) and active crossbar arrays (Li et al., 2018a) have been reported for VMMA. Three-dimensional (3D) stacking of crossbars provides an additional dimension of parallelism, connectivity, and efficiency for complex neural networks (Lin et al., 2020). The DL neural network has many variants and components, for instance, a fully connected layer, convolutional layer, and recurrent neural network. The mapping between the memristive array and computing layers (Yao et al., 2020) is necessary to achieve the designed topology and is crucial for realistic applications.

DL accelerators require both linear current-voltage (I-V) relation and linear weight update characteristics of the memristive synapses. Optimization of material stacks and electrical operation protocols can ensure very stable and linear I-V characteristics for voltage-conductance multiplication (Hu et al., 2018a). The differential pair (G+/G-) method (Suri et al., 2011) has been widely used in both RRAM and PCM synapses, to allow for both positive and negative synaptic weights using electrical conductance, and more importantly to compensate for any asymmetry between the two weight update directions. Other methods are also used to improve the linearity of synaptic weight updates, for instance, multi-parallel devices for mapping the least significant and most significant weight components in a single synapse (Ambrogio et al., 2018).

So far, memristive DL accelerators have experimentally achieved reasonable accuracy in relatively small-scale tasks such as recognition of the image in the Modified National Institute of Standards and Technology (MNIST) and Canadian Institute For Advanced Research (CIFAR) datasets (Yao et al., 2020; Ambrogio et al., 2018). Such demonstrations remain to be seen in larger-scale DL networks like ResNet and other network structures for ImageNet (He et al., 2016).

#### Optimizing Linear Response of the Memristive Device

**Figure 2:**
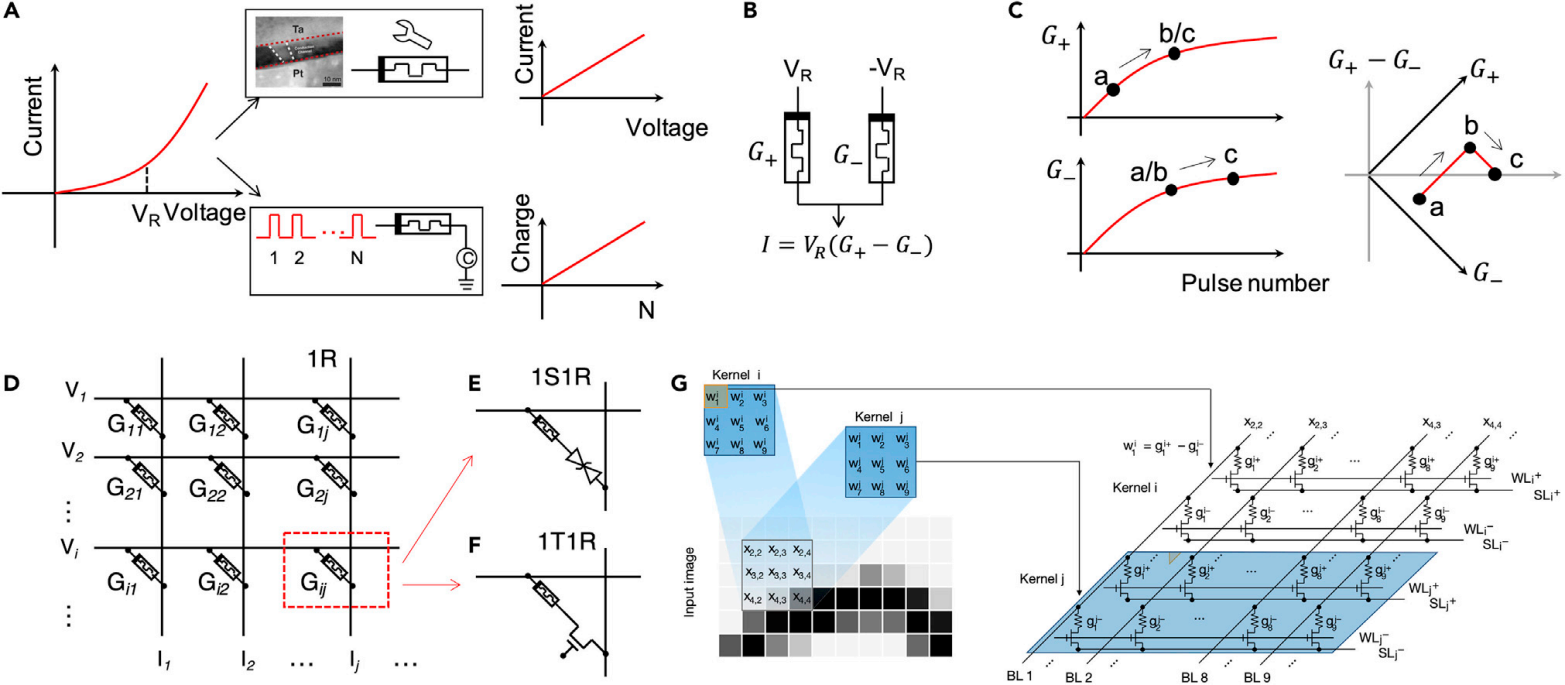
Material- and Device-Level Optimizations to Compensate for the Nonidealities of Memristive Synapses (A) Dealing with non-linear current-voltage (I-V) relation in memristive devices. Left: a typical I-V relation showing exponential dependence of the reading current on applied voltages. Upper right: material modification for linear I-V relation by using composition modulation of localized conduction channels. Reproduced from (Jiang et al., 2016), CC BY. Lower right: pulse number modulation with the number of pulses representing the strength of the input signal and accumulated charge representing the weighted output, resulting in linear behavior between the charge and the pulse number. (B) Differential pair for implementing both positive and negative synaptic weights. (C) Learning errors as the result of the nonlinearity of the weight update. For gradual conductance increases of both G_+_ and G_-_, potentiation is conducted by applying pulses to the G_+_ part (a → b) and depression is conducted by applying pulses to the G_-_ part (b → c). The same numbers of potentiation and depression should cancel each other; however, it results in different overall weight (G_+_-G_-_) between (a) and (c). (D) An array of memristive devices arranged in crossbar structure for VMMA. The voltage vector was applied to the row bars of the memristive array and the current output in each column bar is sensed as the result of Ohm's law and Kirchhoff's current law, $I_{j}=\sum_{i}G_{ij}V_{i}$. (E) and (F) Illustration of each memristive device in series connected with a passive selective device or a transistor, respectively, for access without affected by or affecting other devices. (G) Mapping 2D convolutional kernels to memristive array for convolutional layers. Reproduced from (Yao et al., 2020), copyright © 2020, Springer Nature.

In the inference stage of the neural network, the synapse is essentially carrying out the weight function, that is, scaling the input signal into an output signal. This can be realized by the memristive device through implementing the Ohm's law: *I* = *G·V*, where *G* is the conductance of the memristive device, *V* is the input signal represented by a voltage, and *I* is the output signal represented by the current through the memristive device. However, memristive devices do not always follow Ohm's law with a linear I-V relation. Since the electron transport in a low conductance state often involves mechanisms like tunneling or hopping transport (Ielmini and Zhang 2007), it is common to observe nonlinear (e.g., exponential) relation between current and voltage (Figure 2A), which compromises the accuracy of directly using Ohm's law for multiplication. This issue is more severe in a passive memristive array where a nonlinear I-V characteristic is deliberately adopted to mitigate the difficulty in selectively programming a target device in the passive array (Alibart et al. 2013).

The linearity of the I-V characteristic can be improved by optimizing the stacks of the memristive device thus the switching dynamics rely on the composition modulation of a localized conduction channel rather than a tunnel barrier (Jiang et al., 2016). A smaller read voltage is preferred for linear read operation since even an exponential I-V relation can be approximated by a linear behavior at small voltages, which, however, may decrease the signal/noise ratio of the circuits and potentially degrade the accuracy of the network.

An alternative way to bypass the nonlinearity of the read operation (for inference) is to use a fixed read voltage while the input signal strength is represented by the duration of the read. The output signal will be the accumulated charge of the output current. The duration can be the width of a single read pulse (Cai et al., 2019) or digitalized into the number of identical pulses, namely, pulse number modulation (Yao et al., 2017; Cai et al., 2019). This technique may sacrifice the inference speed. In addition, charge accumulation of the output current requires capacitor-based circuits or additional processes in the digital domain. On the other hand, using the number of identical pulses to represent input signals may simplify or even eliminate the need for analog/digital conversion circuits.

As previously mentioned, the mainstream algorithms for artificial neural networks are based on gradient descent of the synaptic weight to minimize the difference between the actual and the target outputs. This results in both negative and positive values of the synaptic weights. The negative synaptic weight also has its biological basis of inhibitory connections in the biological neural system. A practical way to realize negative synaptic weight is to use a differential conductive pair as shown in *I*=(*G*_+_−*G*_−_)*·V*, where *G*_+_ and *G*_−_ are the conductances of the positive and negative branches of the differential pair, respectively. The minus sign can be easily implemented by Kirchhoff's current law, as shown in Figure 2B. Although this reduces the synaptic density by two times, it has additional benefits such as a better redundancy for precise representation of the synaptic weight. For example, if one of the memristive device in the pair is stuck to a certain conductance and becomes non-responsive, the pair can still be used to represent any synaptic weight by just programming the other devices in the pair to an appropriate value. Negative synaptic weights can also be realized by subtracting one shared “negative” column (reference) from all “positive” columns (Milo et al., 2019).

#### Compensating Device Non-ideality by Online Learning

The synaptic weight can be learned offline by simulating the artificial neural network in traditional computing systems and then transferring the resulting weights to the memristive differential pairs in memristor arrays. This type of weight learning, although consuming a large amount of computational resources on the offline learning platform, is only a one-time cost. To minimize the write errors, it is necessary to utilize iterated program-and-verify steps until the synaptic weight falls into the target range. However, owing to the limited conductance levels and variations of the devices, discrepancies between the original weights and the transferred ones are inevitable. Device failures would also largely degrade network performance (Li et al., 2018).

One solution to compensate for the inaccurate program and device failures would be on-line learning or *in situ* learning. To minimize the output error, the synaptic weight needs to be adjusted according to the gradient descent rule, which can be denoted by Δ*w* = *η·x·δ*, where Δ*w* is the weight changes, *η* is the learning rate, *x* is the input value, and *δ* is the output error caused by this synaptic connection (defined as the partial derivative of the total output error to this node's output, and can be backpropagated from the last layer to all nodes in the network). The output error can be obtained by comparison between the actual output of the memristive network and target output. Then periodical weight update can be performed to the memristive synapses via repeated presentations of input patterns from the dataset. With the output error calculation performed *in situ* in the memristive network, the weight learning partially adapts to the nonidealities of memristive devices (write noise and device failure), thus mitigating the impact of the memristive nonidealities (Li et al., 2018). A similar approach can also be applied to offline training where the weight update in network simulation takes the nonidealities of the memristive devices into consideration, namely, hardware aware training (Gokmen et al. 2019; Joshi et al., 2020). Furthermore, a recently proposed hybrid training scheme takes the advantages of both *in situ* and *ex situ* training methods to efficiently realize a memristor-based neuromorphic system (Yao et al., 2020). The weights are initially learned and transferred to memristor conductance as in conventional *ex situ* training case regardless of device non-ideal characteristics, and only a small part of the weights is trained *in situ* (e.g., the weights of the last fully connected layer) to adapt to present imperfections and recover the system performance. All these realizations of online learning are clear examples of the strength of the device/algorithm co-design, where device nonidealities are mitigated via system-level algorithm optimization.

#### Realizing Linear Weight-Update

Neural network training is a computationally intensive task. Although it can be a one-time cost for some applications, other applications that involve continuous learning or adaption require more frequent re-training of the memristive neural networks. The iterated backpropagation steps in the online training could be a major barrier for training acceleration with memristive networks. It would be ideal if only one write pulse was needed to adjust the memristive synaptic weight in each learning epoch without the need of knowing the current state of the memristive synapse or verifying the updated state. However, this cannot be achieved in most of the memristive devices because their set (increasing the conductance) and reset (decreasing the conductance) switching processes are nonlinear and asymmetric.

A gradual (analog) conductance change upon write pulses during switching can only be achieved in either set or reset operation. RRAM devices can only be gradually reset while the set is abrupt. In contrast, PCM devices can only be gradually set while the reset process is abrupt. As a result, the devices for differential pair composed of RRAM devices are prepared in relatively high conductance states to start with. If potentiation is needed, then the negative conductance branch *G*_−_ will receive reset operating pulses, whereas for depression, reset pulses will be applied to the positive branch *G*_+_. The opposite configuration can be used for a PCM differential pair. However, in both cases, the amount of the conductance change per write pulse is not constant, rather it depends on the current conductance state of the devices, thus leading to substantially non-linear weight update.

Saturation of conductance change in the low-conductance range of RRAM device during the reset operation and in the high-conductance range of PCM during the set operation is generally observed (Figure 2C). During training, numerous weight updates in potentiation and depression should mostly cancel each other and result in only a small amount of net conductance change. The nonlinear changing of conductance in *G*_−_ and *G*_+_, however, prevents such cancellation in the weight updating process and acts as the largest source of accuracy loss in online training (Burr et al., 2015).

To linearize the conductance change, material- and device-level optimization has been performed. For instance, by inserting an AlO_x_ barrier layer, a HfO_2_-based RRAM device shows an improved linear potentiation (or depression) behavior of conductance under identical pulses (Woo et al., 2016). Choi et al. limited the conductance changes in a smaller window in Ag^+^-based memristive devices by modifying the potentiation pulses to only allow a mild amount of Ag participant in the switching dynamics (Choi et al., 2018). A three-terminal memristive device with an electrochemical gate layer can also make the change of source-drain conductance linear with the pulses applied in the gate terminal (Tang et al., 2018; van de Burgt et al., 2017). These electro-chemical memristive devices, however, suffer from long pulse duration hence a low operational speed. The gradual linear conductance tuning in both directions also allows the potentiation and depression in both sides of the differential pair, avoiding freeze-out of the conductance tuning in unidirectional update (Ielmini and Pedretti 2020).

Capacitor-based synapse has an ideal linear weight update ability but is volatile. A synaptic configuration composed by a major pair of non-volatile PCM devices (G+, G-) and a minor pair of volatile capacitor-based synapses (g+, g-) can thus integrate the advantages of these two parts and lead to linear weight update and non-volatile synaptic weight storage. The minor pair updates its weight frequently in each training cycle and transfers the weight to the major pair periodically. Thanks to the linear weight update ability, a training accuracy comparable with that of a software-based solution can be obtained (Ambrogio et al., 2018). However, this solution results in a bulky individual synapse (2PCM + 3T1C), which occupies a relatively large area on the silicon chip.

#### Utilizing Memristive Arrays for VMMA

Mapping the fully connected layers of artificial neural networks to the two-dimensional (2D) memristive arrays is straightforward since the computation in the fully connected layers are essentially VMMA operations (Ielmini and Pedretti 2020), $y_{j}=\sum_{i}w_{ij}x_{i}$, where *i* and *j* denote the indices of the input vector and output vector, respectively. It can be directly implemented by applying voltages to the rows of a memristive array and sensing the current output at the end of each column as the result of Ohm's law and Kirchhoff's current law, as shown in Figure 2D.

Note that the case shown in Figure 2D illustrates the ideal case of using a passive memristive array (Prezioso et al., 2015). However, there is a dilemma in a passive array between the desire for a linear I-V relation to directly utilize Ohm's law during inference and the desire for a nonlinear I-V relation to suppress the sneak path currents and program individual devices during learning. Adopting a selector in each cell to form the so-called one-selector/one-resistor (1S1R) structure (Figure 2E) increases the nonlinearity for array programming, which, however, makes the pulse duration/number modulation scheme discussed before an only suitable choice for the inference.

A more practical implementation of VMMA is to use an active array where each cell of the array is composed of a memristive device and a transistor connected in series (Figure 2F). With such transistors in the array, an individual memristor can be selected by activating only the corresponding transistor(s) for reading or programming operations without affecting other devices in the array. With a high on/off ratio of the mature transistor technology, a large memristive crossbar array can be achieved, with the only limitation essentially being the wire resistance. Fully parallel reading during inference can be enabled by turning on all the transistors in the array, and semi-parallel programming (e.g., row by row) is also possible. The cell size is limited by the transistor footprint, which results in a lower synaptic density than the passive crossbar array. The overall area efficiency has been limited by the peripheral circuits for neuronal functions (section “Co-design with CMOS Peripheral Circuits”) in most hardware implementations of memristive deep networks so far. When innovative designs are devised to significantly reduce the area of such peripheral circuits, the 1T1R synaptic arrays limited by the size of the transistor will then become the primary concern of the area efficiency.

The parasitic effects existing in the array also limit the size of the array. For instance, when the wire resistance is comparable with the device resistance, the voltage drops on the wire connections lower the real voltage applied to the memristive device and might result in program or read errors, i.e., IR drop issues. The actual limit of the size of an array is affected by the wire resistance, the on resistances of the devices, and the on/off ratios. Assuming the memristive device has resistances of 100 kΩ and 10 MΩ in the low-resistance state and the high-resistance state, respectively, and taking the Cu wire interconnection resistance parameter from the ITRS roadmap, Zuloaga et al. predicted that array size up to 256 × 256 can work well after being downscaled to 10 nm technology node (Zuloaga et al., 2015), which is sufficient for most of the demonstrations of deep networks reported so far. If a larger array size for VMMA operation is needed, material-level optimizations to decrease the resistance of the connection wires or to increase the resistance of the memristive devices are needed (Zhang et al., 2019). Alternatively, system reduction schemes that can substantially reduce the weight matrix size can be utilized (Liu et al., 2014). It is also possible to mitigate the IR drop by a compensation scheme retraining the dominant neurons (Jeong et al., 2018). Online adaptive learning (Li et al., 2018), where the automatic weight update will adapt to the mismatch of the actual cell resistance/conductance with the desired synaptic weight, can also mitigate the parasitic effects in array configurations.

Convolutional neural networks are more suitable for recognizing static 2D inputs, such as images, thanks to the biologically inspired convolutional kernels similar to the receptive field of complex cells in the visual cortex. Mapping the convolutional layers to memristive arrays needs first converting the convolutional operation of matrix-matrix multiplication of each kernel and its receptive field to a vector-vector multiplication (Gao et al., 2016). The kernel vectors are aligned in columns or rows of the memristive array, and image input is partitioned in receptive fields (patches) and sequentially fed to the kernel vectors, as illustrated in Figure 2G (Gokmen et al., 2017; Yao et al., 2020). This weight sharing methods by the convolutional kernels is also named as spatial weight sharing (Wang et al., 2020c). Spatially shared memristive weight strongly reduces the number of memristive devices needed for a deep CNN. For comparable accuracy of recognizing handwritten digits from MNIST database, the three-layer fully connected neural network consumes 329,770 PCM devices (accuracy 97.94% [Ambrogio et al., 2018]), whereas the five-layer convolutional neural network uses 5,629 RRAM devices (accuracy 96.19% [Yao et al., 2020]).

Spatially shared kernel vector weight, however, requires repeated vector-vector multiplication between input image patches and the convolutional kernel. The number of patches increases quadratically with the image size (Gokmen et al., 2017), which reduces the throughput and becomes the bottleneck of the system performance. It is also possible to parallelly implement the convolutional operation in fully connected topology (Lin et al., 2020) or partially reduce the spatial share factor. Replicating and transferring identical convolutional kernels to multiple 2D memristor arrays provides a possible solution to boost the parallelism of convolution operations and enhance the throughput accordingly (Yao et al., 2020). However, a careful trade-off between the inference speed and memristive area cost is necessary.

Memristive devices are suitable for 3D integration, a powerful solution for high-density storage, and most importantly, for enhancing the neuronal connectivity required in complex neural networks. The 3D integration enables parallel and faster convolutional calculation for neuromorphic application (Huo et al., 2020; Lin et al., 2020), bypassing the trade-off between the inference speed and memristive area. As the cross-section of the 3D array is a 2D interface to directly accept 2D image inputs, the image patches and convolutional kernel matrices no longer need to be unrolled to vectors (Lin et al., 2020). Moreover, the additional dimension could enable massive connections and increase the flexibility of memristor topologies. By defining the sliding kernels at each patch as zigzag staircases in 3D space and shaping both the top electrode and bottom electrode as vertical pillars, input signal could be fed into different patches through the same pillar simultaneously. In this manner, the whole image could be presented at the same time and the 3D device structure enables all convolutional operations during the sliding procedure to be processed in parallel, saving the sequential shifting time and improving the system performance. Additionally, unrolling 3D convolutional kernels to 2D matrices, a 3D convolutional neural network for stereoscopic object recognition can be realized (Huo et al., 2020).

Other variants of the DL neural network, like long short-term memory (LSTM) (Li et al., 2019; Wang et al., 2019c) and deep belief neural network composed by RBMs (Eryilmaz et al., 2016), can also exploit the benefits of VMMA capability of memristive arrays, which is very attractive as VMMA consists the major part of the computations in these networks. However, other essential functionalities, like the gate unit controlling the memory time for LSTM cells and probability generations in RBM neurons, are carried out in software. These functionalities can also be implemented by exploiting the unique features of memristive devices more morphologically, which will be covered in the next section.

#### Co-design with CMOS Peripheral Circuits

The program and read operations on the memristive array and the weight update calculation need to be carried out by complementary metal–oxide–semiconductor (CMOS) circuits mimicking the behavior of biological neurons, which should be integrated closely with the memristor array on the same chip to further enhance the efficiency. Tailored according to the targeted application and specific hardware architecture, the generally utilized circuitry blocks could include the sample-and-hold (S&H) module and analog-to-digital converter (ADC) to temporarily hold the summed analog currents and transform them to the digital domain, respectively. The digital-to-analog converter (DAC) that converts digital inputs into appropriate voltage amplitudes should be counted if a voltage amplitude-encoding scheme is adopted. Digital control, processing, and routing blocks are also necessary to realize activation functions and monolithically integrate a complete neuromorphic system (Shafiee et al., 2016; Hu et al., 2018). Furthermore, extra peripheral circuits need to be considered to realize various kinds of on-chip learning rules. It is worth mentioning that in practical system implementation, the bottom-level device characteristics and the top-level algorithm optimizations would jointly determine the circuit and architecture design to meet the necessary hardware performance. The array size, the precision and speed of ADCs, and other circuit aspects need to be carefully considered with trade-offs between hardware efficiency and cost.

CMOS implementation of the peripheral circuits can result in a large area and power consumption. For instance, the DAC and ADC circuits can occupy a much larger area (e.g., 21.57 mm^2^) than the dense synaptic array (e.g., 0.14 mm^2^) (Cai et al., 2019). A commonly used strategy to improve the system area efficiency is to temporally share the DAC and ADC elements. State-of-the-art high-precision ADCs consume large area; however, they can have high sampling rates. Thus multiple output nodes of the memristive array can share a single ADC sequentially (Gokmen and Vlasov 2016). An additional multiplexer (MUX) circuit is needed for the sequential selection of output nodes. ADCs of 6–8 bit precision are needed for acceptable accuracy loss of neural networks with a relatively small size while further lowering the precision may induce high accuracy deterioration (Li et al., 2016). Nevertheless, the required precision for large-scale neural networks depends on the exact network structure or dataset. Further reducing the precision can be achieved by separately handling the outlier values and normal values with 4-bit ADCs, showing a relatively small loss of performance (Park et al. 2018). A binary neural network is a possible option to address the area and power inefficiency in ADC- and DAC-based neuronal function realizations, which will be further discussed in section “Exploiting Bistable Behavior of Memristive Devices.” It is also possible to use neurons working directly in the analog domain (Krestinskaya et al., 2018), thus eliminating the need for the conversion between analog and digital signals. This requires the analog neuronal circuit to perform (in the analog domain) activation functions, like sigmoid or ReLU, which, in most of the hardware deep neural network demonstrations so far, have been implemented by controlling computers or microcontrollers in the digital domain after the conversion of the signal from the analog domain. Using integrate&fire neurons to convert the output of VMMA to spiking trains, where the spike count or frequency denotes the analog value of the neuron output, can also eliminate the need of analog to digital conversion (Yan et al., 2019).

For a large-scale integration of the synaptic and neuronal components with the learning algorithms in a practical system, utilizing the above strategies, in-memory computing macros or neuromorphic computing macros have been proposed, such as ISAAC (Shafiee et al., 2016), PRIME (Chi et al., 2016), and Pipelayer (Song et al., 2017). These macro circuits can be tiled together according to the structure of deep neural networks to be constructed. For a more comprehensive review of peripherical circuits and large-scale integration, readers can refer to Yan et al., 2019. These memristive DL accelerators are projected to be superior to CMOS-based or other solutions in several aspects, such as in performance (operation per second, OPS), area, and power efficiency (Zhang et al., 2020a; Sebastian et al., 2020). Unfortunately, ideal switching characteristics and linear I-V characteristic are assumed in these designs. In terms of the chip-level demonstration, a fully integrated memristive in-memory computing macro, named as non-volatile computing-in-memory (nvCIM), has been demonstrated (Chen et al., 2019). However, the nvCIM macro works on the binary-input ternary-weight model and will not fully exploit the analog in-memory computing ability of a memristive array. The realization of activation and intra-layer communication is carried out by off-chip field-programmed gate array (FPGA). Recently, this field has been rapidly developing toward monolithically integrated memristive neuromorphic systems, even though the memristive analog behavior has not been fully exploited (Liu et al., 2020; Wan et al., 2020).

In Table 1, we summarize the optimization and design efforts in various levels of memristive neuromorphic computing with the purpose of DL accelerators. The color code refers to the degree of optimization/co-design of each implementation. Despite the numerous efforts in Table 1, a general-purpose memristor accelerator for general neural networks is still missing, partially because device reliability and uniformity issues across multiple arrays are yet to be solved. On the other hand, conventional DAC and ADC solutions consume a large area and energy, becoming the bottleneck of system performance (Cai et al., 2019). Novel routing schemes with the least requirement for the on-chip memory are also needed to make the most of the memristor neuromorphic system.

**Table 1:** A Survey of Optimization and Design Considerations in Various Levels of Memristive Neuromorphic Computing

NA: not applicable (or not discussed).

^a^ Training within a transfer interval was performed in software with device models and read PCM devices are operated when transfer needed.

^b^ Data retrieved from (Li et al., 2018).

^c^ Projected for all peripheral circuits integrated on chips with 128 × 128 array and four columns of the memristive array sharing one ADC converter.

^d^ ML-CSA: multi-level current-mode sense amplifier; DR-CSA: distance racing current-mode sense amplifier.

### Exploiting Memristive Properties for Brain-Inspired Algorithms

In addition to serving as a static memory of synaptic states for in-memory-computing in deep learning algorithms, memristive devices also have a variety of dynamical properties that share close similarities with biological components, which can potentially lead to computing with augmented efficiency and intelligence. Novel brain-inspired learning algorithms are needed to utilize these intrinsic properties of memristive devices, e.g., the stochasticity of the state (Yu et al., 2013), the dynamics of state transition, and second-order effects (Du et al., 2017).

#### Exploiting the Stochasticity

Various random physical phenomena exist in memristive devices, resulting in stochastic variations of conductance levels and switching parameters. For instance, for RRAM and PCM devices, owing to the nature of the ionic-electronic coupled transport mechanism, intrinsic stochasticity exists as random telegraph noise in the reading phase and variation of switching parameters in the weight update phase (Carboni and Ielmini 2019). Stochasticity is a critical problem for memory and storage applications and their usage as synaptic weights in DL accelerators. However, stochasticity as a physical entropy source can be exploited for generating true random numbers or physical unclonable functions for information security. Additionally, they can provide a low-cost solution for implementing some specific neural network algorithms where stochasticity is essential for computation.

**Figure 3:**
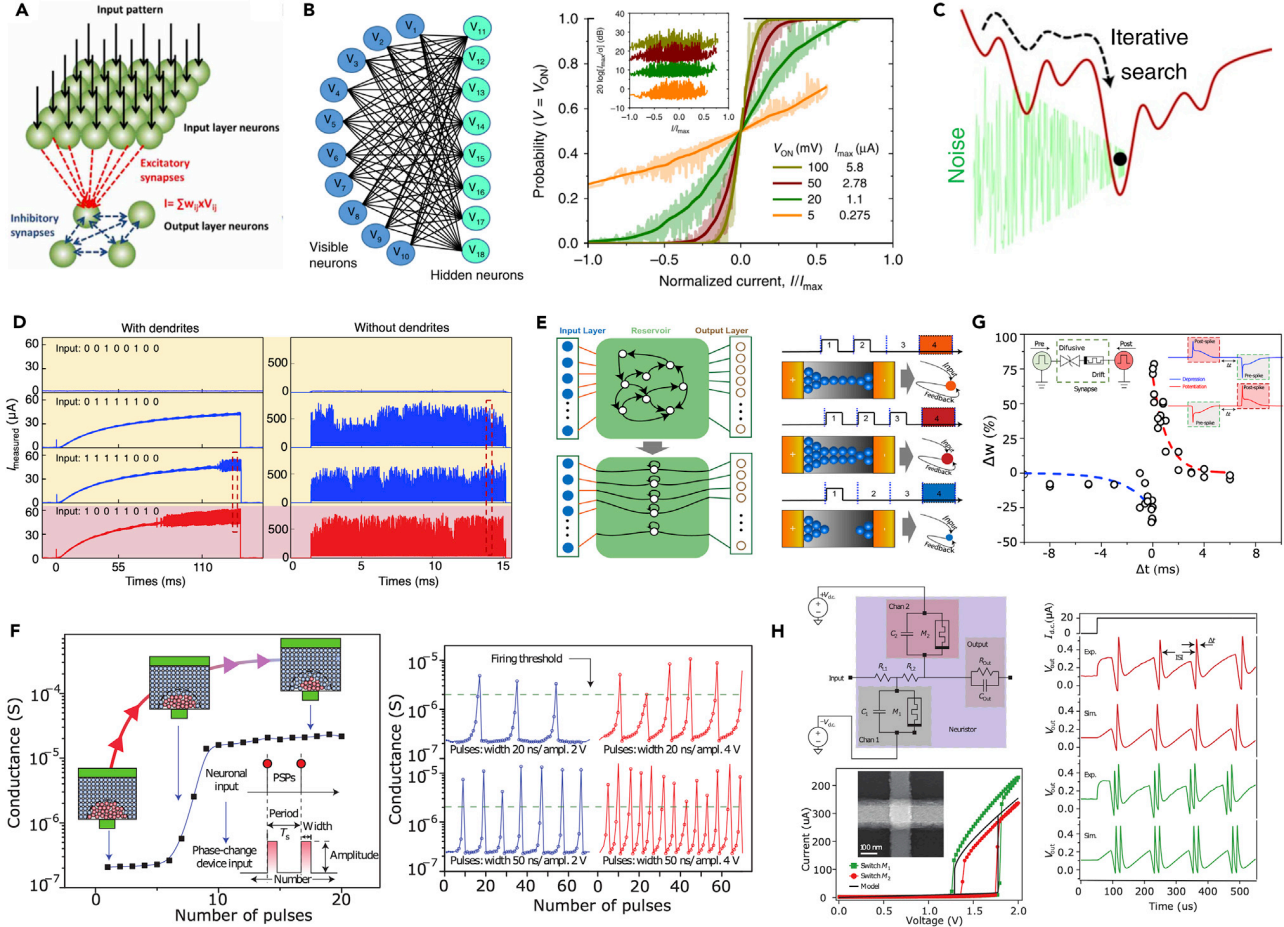
Various Bioinspired Algorithms Exploiting the Unique Features of Memristive Devices (A) A winner-take-all neural network exploiting the stochasticity of the memristive devices. Reproduced from (Yu et al., 2013), CC BY. (B) A restricted Boltzmann machine exploiting the intrinsic reading noise of the memristive array. Reproduced from (Mahmoodi et al., 2019), CC BY. (C) Exploiting the intrinsic noise of memristive array to avoid trapping in local minimum sites in a Hopfield memristive neural network. Reproduced from (Cai et al., 2020), copyright © 2020, Springer Nature. (D) Memristive dendrites exploiting the non-linearity of memristive devices for filtering and integration functions. Reproduced from (Li et al., 2020), copyright © 2020, Springer Nature. (E) Reservoir computing realized by the short-memory effect of diffusive memristors. Reproduced from (Midya et al., 2019), CC BY. (F) A PCM device-based neuron with the gradual set switching of the PCM device was used to mimic the integration function of neurons. Reproduced from (Tuma et al., 2016), copyright © 2016, Springer Nature. (G) Spike-timing-dependent plasticity (STDP) enabled by the short-term memory (volatility) of the diffusive memristive device. Reproduced from (Wang et al., 2017), copyright © 2016, Springer Nature. (H) A Mott transition memristor device-based Hodgkin-Huxley neuron faithfully reproducing biological spike shape and tunable spiking trains. Reproduced from (Pickett et al., 2013), copyright © 2013, Springer Nature.

Under a weak programming condition, the set transition of metal-oxide memristive devices becomes probabilistic. A winner-take-all network can be realized by the competition among post-neurons utilizing the probabilistic switching in synapses (Yu et al., 2013), as illustrated in Figure 3A. In RBM, the sampling and reconstruction stages heavily rely on the probabilistic of hidden or visible units being activated. A dot product circuit incorporating the stochasticity coming from the intrinsic noise of the memristor array for the RBM has also been demonstrated (Mahmoodi et al., 2019). Using a passive memristor crossbar, a single-layer RBM with ten visible and eight hidden neurons is demonstrated with the energy function minimization (Figure 3B). In another work of implementing the Hopfield neural network, the intrinsic noise of a memristive crossbar was used for a combinatorial optimization problem (Cai et al., 2020). A moderate noise level was found useful for the network to escape from local minimum points in the energy landscape better than both the noise-free and the high noise level situations (Figure 3C).

The stochasticity of magnetic tunnel junctions (or MRAM) was reported to implement three-terminal probabilistic bits (p-bits) (Borders et al., 2019). These p-bits can be viewed as probabilistic neurons that are electrically connected to form an asynchronous network for factorizing integers up to 945 (63 × 15) adapting diabatic quantum computing algorithm. Tunable probability of the random switching of the superparamagnetic tunnel junction also allows the population coding where each neuron embodied by a superparamagnetic tunnel junction is associated with a specific range of inputs, which is then computed as a weighted sum of the rates of each neuron (Mizrahi et al., 2018). Similar tunable probability can be achieved by utilizing the inherent random noises of analog RRAM devices and was used to construct a Bayesian inference neural network that shows high resilience to adversarial testing samples (Lin et al., 2019).

#### Exploiting the Current-Voltage Non-linearity

I-V non-linearity of memristive devices could be a major issue when the devices are used as synapses for DL accelerators. However, such non-linearity is an essential synaptic or neuronal behavior in cellular neural networks (Duan et al., 2015; Caravelli et al. 2017), where it enriches the dynamics of the system. It has also been shown that the non-linearity can be used to mimic the non-linear integration of biological dendrites (Lavzin et al., 2012), constructing a memristive dendrite (Li et al., 2020).

In conventional algorithms of DL neural networks, neurons act as simple elements summing all inputs from synapses. It is found in neuroscience that the non-linear integration of synaptic signals by the dendrites provides primitive processing before the signals reach the neuron body (Agmon-Snir et al., 1998). Recently, a memristive dendrite component has been demonstrated using a Pt/TaO_x_/AlO_δ_/Al-based dynamic memristor (Li et al., 2020), exploiting the non-linearity provided by the Schottky-like barrier in the Pt/TaO_x_ interface. By adding non-linearity before the neural summation to realize the non-linear dendritic function can further enhance the performance of the neural network. With memristive dendrites filtering the background signals, the spiking output of the neuron shows more distinction between false patterns and true pattern (Figure 3D), and, at the same time, reduces the power consumption. Thus, performance enhancement in both energy efficiency and accuracy is obtained with the addition of non-linear memristive dendrites.

#### Exploiting the State-Variable Accumulation

Upon stimulation of weak electrical pulses, the memristive devices sometimes do not show explicit conductance changes. However, explicit conductance may be induced by subsequent pulses only if there are prior pulses. This behavior happens because of the internal state-variable, for instance, the temperature being an internal memory for historical stimuli (Kim et al., 2015). The accumulation of the state-variable may offer an internal timing mechanism and enables an activity-history-dependent modulation of the first-order state, namely, conductance; thus, is also called second-order effect. It has been used to mimic the effect of the Ca^2+^ dynamics of biological synapses and enables the temporal learning of timing-encoded information (Zidan et al., 2017).

In Ag-based diffusive memristors, the configuration of the transporting ions in the dielectric layer before the threshold switching shares a similar behavior. The accumulation of ions before their final formation of a continuous filament bridging the electrodes can act as internal memory for historical stimuli. The drift of Ag ions under electrical stimuli and the diffusion of Ag ions under zero electrical bias faithfully emulate the ion dynamics that play a critical role in the neuromorphic functions of biological intelligent systems (Wang et al., 2017). For instance, pair-pulsed facilitation and depression were found in the diffusive memristor-based synapse for a high frequency of pulses and low frequency of pulses, respectively (Wang et al., 2017), which also enables the temporal learning naturally.

Reservoir computing (RC) can offer efficient temporal processing of recurrent neural networks with a low training cost. RCs based on the second-order effect (Du et al., 2017; Moon et al., 2019) and the accumulation of ions in diffusive memristors (Midya et al., 2019) have been explored. The RC exploiting the accumulation or integration of the state-variable (secondary internal variable or ion configurations) acts as a framework extracting features from temporal inputs. Taking advantage of the rich short-term dynamics of the diffusive memristive device, an RC system is constructed with one reservoir layer of a diffusive memristors and one readout layer of a nonvolatile memristor-based trainable perceptron neural network, with which classification of temporally rearranged handwritten digits from the MNIST database is achieved with a much-reduced training workload, as shown in Figure 3E (Midya et al., 2019). Without the short-term dynamics in the state-variable accumulation, neural networks for processing temporal information should have additional memory gate to control the learning and forgetting of the historical information, resulting in extra costs in circuitry and energy.

The state-variable accumulation upon electrical stimuli can be used for the integration function of an artificial memristive neuron (Tuma et al., 2016). The gradual set of PCM device has been utilized to demonstrate an artificial neuron capable of integrating post-synaptic potential at the nanoscale, where the phase configuration (thus the conductance) of the nanoscale PCM device represents the membrane potential, as shown in Figure 3F (Tuma et al., 2016). With the gradual internal state change upon pulses mimicking the integrating function and its consequential abrupt switching representing the fire behavior, a silicon oxide RRAM cell is reported to emulate a biological neuron (Mehonic and Kenyon 2016). A similar function can also be realized by the accumulation of ion transport in a diffusive memristor (Hao et al., 2020). This approach results in a capacitor-free version of a solid-state neuron; however, it requires a reset of the memristive device back to its original state after each fire.

#### Exploiting the Volatile Memristive Switching

Industrial memory storage application of memristive devices requires that the device can retain its conductance state for at least 10 years; thus, it is also called non-volatile memory. In neuromorphic computing for DL accelerators, a similar requirement should be fulfilled, that is, the memristive conductance for synaptic weight should remain stable for a long time to preserve the learned knowledge. However, some memristive devices with Ag as one of its electrode shows short retention time for the high conductance state (Bricalli et al., 2018; Wang et al., 2019b). The retention time is usually reported to be in the range of sub-microseconds to tens of milliseconds. This can be viewed as short-term synaptic plasticity and is reported to demonstrate some time-related computing functionalities (Wang et al., 2018a). The volatilities can be modulated by the strength of the stimuli. An increase in the frequency of applied pulses (Ohno et al., 2011) or using a higher compliance current (Wang et al., 2019b) can cause a transition from volatile to non-volatile memory, corresponding to the short-term plasticity and long-term plasticity, respectively.

The short-term plasticity of volatile memristive device allows the STDP learning with non-overlapping spikes to be demonstrated in a combined synapse of one volatile memristive device and one non-volatile memristive device (Wang et al., 2017), with the finite delay time of the volatile memristive device bridging the time gap of the non-overlapping spikes (Figure 3G).

Another approach for memristive neurons is to utilize the abrupt and volatile switching of memristive devices for the fire functionality (Wang et al., 2018d; Zhang et al., 2018), whereas the integration function is completed by charge accumulation in an external or parasitic parallel capacitor or internal state accumulation before the abrupt switching (Zhang et al., 2018b). The volatility of the memristive device enables the artificial neuron to recover its resting state spontaneously after the abrupt switching on, which is obtained by a device reset operation after each firing event in the nonvolatile memristive neurons discussed in section “Exploiting the State-Variable Accumulation.” Owing to the simple structure and nanoscale-level scalability, these memristive neurons can be much more compact than the bulky CMOS neurons. Moreover, in case long time constants, such as tens of milliseconds, are needed to match the normal time constants of the biological systems, huge capacitors would be required in the CMOS neurons (Qiao et al., 2015). In contrast, a nanoscale diffusive memristor would readily provide such time constant. The implementation of memristive neurons has also enabled fully memristive neuromorphic computing (Wang et al., 2018), further enhancing the integration level of the hardware neuromorphic computing.

Volatile memristive switching sometimes accompanies negative differential resistance arising from an insulating-to-conducting phase transition or Mott transition, namely, Mott memory device (del Valle et al., 2019; Zhang et al., 2020b). Using two Mott memristors with transient memory as ionic channel and two capacitors as charge storage, a neural circuit named as neuristor was built as a hardware Hodgkin-Huxley model (Hodgkin and Huxley 1952) that faithfully mimicked the action potential generation in biological axons, as shown in Figure 3H (Pickett et al. 2013). In another work, more biologically plausible and intrinsically stochastic neurons were built with vanadium dioxide Mott memristors, which exhibited twenty-three types of biological neuronal behaviors (Yi et al., 2018). The controllable frequency of spikes in these artificial neurons also finds applications in coupled oscillator networks (Csaba and Porod 2020).

#### Exploiting Bistable Behavior of Memristive Devices

Without fine material-level and device-level optimization, the memristive device usually shows limited conductance levels other than the capability of analog conductance tuning. With limited conductance states, the conventional artificial neural network needs to be adapted. This can be done by quantizing the analog weight value from offline learning (Milo et al., 2019), which generally results in some loss of recognition accuracy. Many memristive devices only show binary stable states, i.e., high conductance state and low conductance state. For memristive devices embodied as STT-RAM and FeRAM, analog switching is generally more challenging.

To exploit the bistable behavior of memristive devices for synaptic applications, a binary neural network was proposed relying on binary synapses (only with two states) and binary node value (Hirtzlin et al., 2020). In the binary neural network, since the weights and inputs from the preceding layer are both binary valued, the weighted outputs are also binary, thus the vector-matrix multiplication becomes an XOR operation (Luo et al., 2019). The accumulation/summation function in the neural nodes degenerates to POPCOUNT operations, i.e., counting the number of “1”s in a series of bits, eliminating the needs of a high-precision current sensor. The activation function afterward is only a sign function, further reducing the computational needs in the neuron nodes. The binary neural network also shows high tolerance to weight bit error (Hirtzlin et al., 2020).

Ternary content-addressable memory (TCAM) is another algorithm that intrinsically exploits the bistable behavior of memristive devices (Yang et al., 2019; Ni et al., 2019). TCAM can perform in-memory search and pattern matching between the query feature vector and stored vectors of binary bits. In the study by Yan et al., 2019a, Yan et al., 2019b, 2-transistor/2-RRAM TCAM cells were used to store the TCAM vectors. For each TCAM cell, the stored TCAM datum was defined as the bit “1” for RRAM1 in HRS and RRAM2 in LRS, the bit “0” for RRAM1 in LRS and RRAM2 in HRS, the bit “X” (do not care bit) for both RRAMs in HRS. Thus, only two states of the memristive device were required. Within a similar scenario, ferroelectric TCAM with each cell only consisting of two ferroelectric field-effect transistors (FeFETs, three-terminal forms of FeRAM) has also been proposed (Ni et al., 2019). Recently, an analog memristive TCAM was introduced by taking advantage of the analog programming in RRAM devices (Li et al., 2020a).

### Toward a Brain-like Computing System

**Figure 4:**
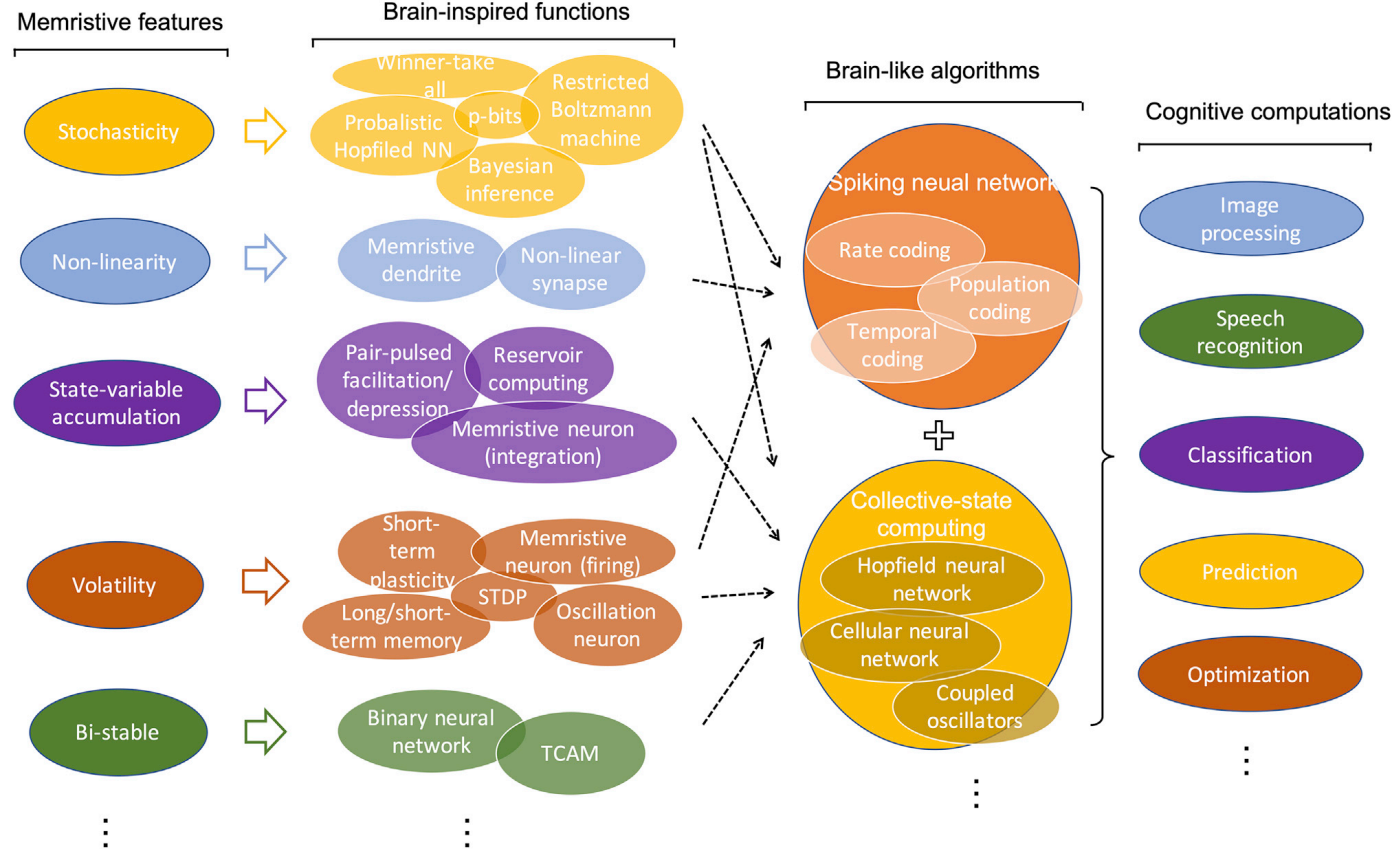
Exploiting Memristive Features in Various Brain-Inspired Algorithms and Projections of Using These Brain-Inspired Functions for Cognitive Computations with Brain-Like Algorithms The unique features of memristive devices have been proposed to realize various brain-inspired functions for a neural network (from the first column to the second column); however, how to combine these brain-inspired functionalities to realize brain-like algorithms (from the second to the third) and for practical cognitive functions (from the third to the fourth) has no clear paths.

The first and second panels of Figure 4 summarize the projections of various memristive features and the corresponding brain-inspired functions discussed in previous section. Building upon these components, the next step would be the construction of brain-like algorithms and realization of cognitive computations as alternative solutions to the DL techniques. Mainly, two approaches can be seen in recent developments of memristive neuromorphic systems. One is from the signal morphological aspect, to emulate the spiking behavior of the biological neural network (Roy et al. 2019). The other one is from the connection morphological aspect, to emulate the collective state dynamics and evolution of the biological network. The SNN (Wang et al., 2018b), closely mimicking the information presentation in biological neural systems, is considered as a viable way to achieve brain-like computing with high energy efficiency and error tolerance. Collective-state computation has several forms, such as Hopfield neural network (Hopfield 1982), cellular neural network (Chua and Yang 1988), and coupled oscillators (Csaba and Porod 2020), mimicking brain activity at a high level, solving the problem by the system automatically finding its stable states in its energy landscape. A hybrid solution of these two approaches is also possible. However, there are no clear projections of which and how many brain-inspired functions can be utilized in these two approaches as illustrated in Figure 4.

#### Spike-Timing Dependent Plasticity and Spiking Neural Network

SNN is considered as the third generation of neural networks (Maass 1997), following the first generation based on McCulloch-Pitts neurons (McCulloch and Pitts 1943) with digital input and output, and the second generation composed of multiple perceptron layers with gradient descent learning algorithm, which applies activation functions after the weighted sum of the inputs and achieves analog-valued input and output (Lecun et al., 1998).

The various DL neural networks with analog-valued input and output in section “DL Accelerators by Memristive Hardware” can be in general converted to an SNN, with the spiking rate of each neuron proportional to the analog value. Instead of ADC/DAC conversion introduced in “Co-design with CMOS Peripheral Circuits”, the analog current/voltage in the memristor array can be converted by integrate and fire (IF) neurons (Milo et al., 2016; Yang et al., 2019). However, this does not fully exploit the benefits and capabilities of an SNN. In the human brain, by encoding information using spike timing, an extremely sparse and energy-efficient representation can be achieved (VanRullen et al. 2005). Conversion of the analog current/voltage in the memristor array into spatial-temporal spike representation in the digital domain using leaky integrate and fire (LIF) neurons with temporal dynamics (Fang et al., 2019) provides the possibility of constructing spatiotemporal spiking neural network.

Instead of error backpropagation, one commonly utilized mechanism for learning in SNNs is the STDP of synapses (Bi and Poo, 1998). The STDP learning rule has its biological root originated from the Hebbian learning rule, where “neurons that fire together, wire together” (Hebb 1949). Memristive synapses capable of STDP and triplet-based learning have been widely reported (Wang et al., 2015, 2020b). The weight updates depend on the timing of the presynaptic and postsynaptic spikes: the synapse weight is potentiated if the presynaptic spike precedes the postsynaptic spike, and depressed otherwise. The general realization of this STDP property in memristive devices is based on the engineered shapes of the presynaptic spike signal and the postsynaptic spike signal and their overlap in time (Linares-Barranco and Serrano-Gotarredona 2009; Stoliar et al., 2019). It can also be realized without spike overlapping by utilizing the internal dynamics of volatile diffusive memristors, which faithfully emulate what happens in biological synapses (Wang et al., 2017) (see also section “Exploiting the Volatile Memristive Switching”).

**Figure 5:**
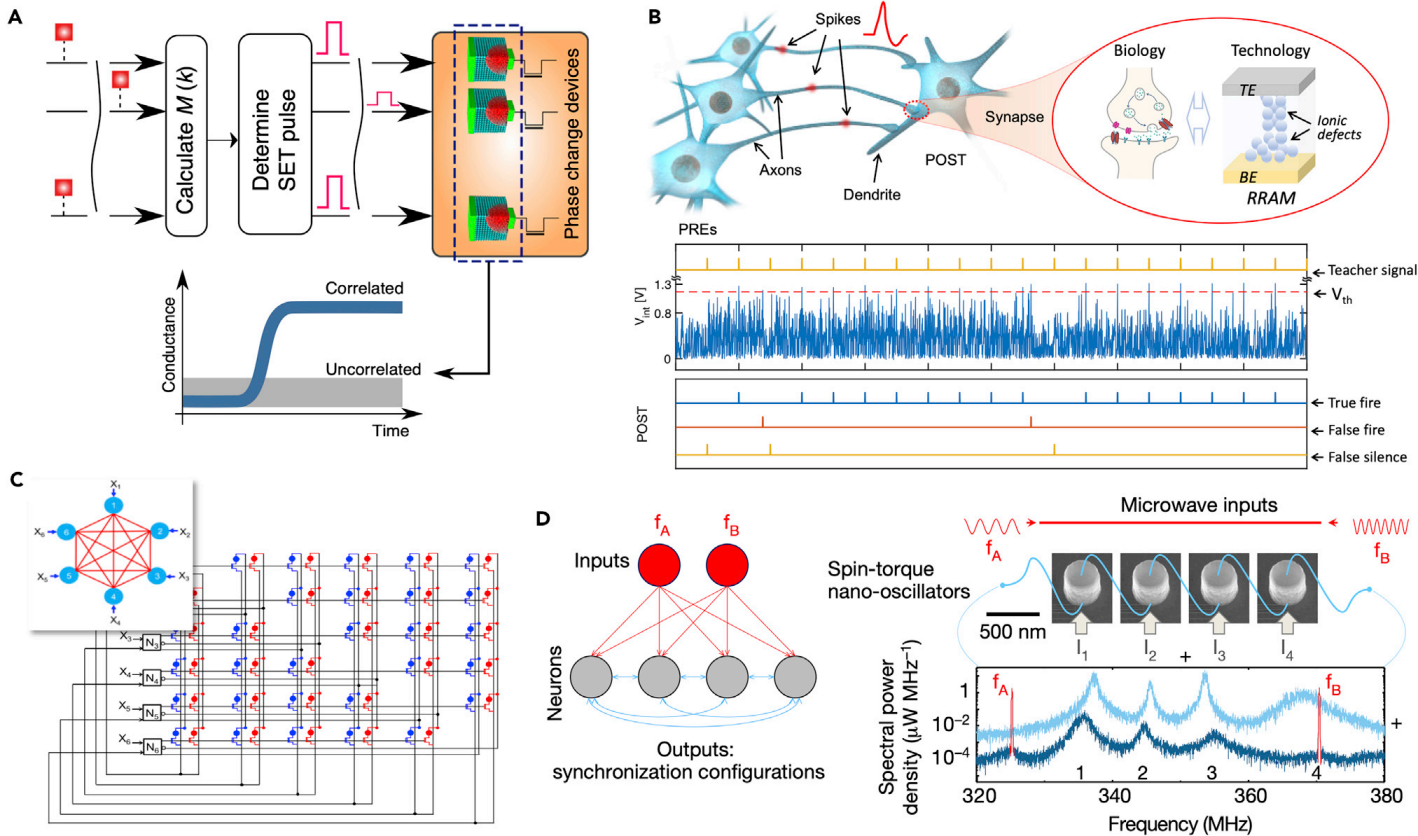
Construction of Memristive Neuromorphic System Utilizing Brain-Inspired Algorithms Enabled by Memristive Devices (A) Learning and recognition of an image by the detection of the coincidence of simultaneous spikes with the help of PCM synaptic devices capable of the STDP. Reproduced from (Sebastian et al., 2017), copyright © 2016, Springer Nature, CC BY. (B) Spatiotemporal computation considering the precise times of each spike within a spiking pattern. Reproduced from (Wang et al., 2018b), CC BY. (C) A Hopfield neural network for associative learning by fully connecting all neurons via bidirectional synapses. Reproduced from (Milo et al., 2017), copyright © IEEE 2017. (D) Coupled nano-oscillators enabling mimicking of neural synchrony for vowel recognition. Reproduced from (Romera et al., 2018), copyright © 2018, Springer Nature.

Several neuromorphic systems based on the STDP weight update mechanism have been reported for pattern recognition. A PCM-based one-layer neural network for online pattern learning and recognition has been demonstrated by assuming the alternation of pattern and noise spikes from the pre-neurons and competition between post-neurons (Ambrogio et al., 2016). The essential idea is that the simultaneous pattern spikes in the pre-neurons result in a spike in one of the post-neurons, and potentiation will be induced in their connecting synaptic devices via the STDP rule, while noise spikes following the spike of the post-neuron result in depression of the according synaptic devices. The same methodology can be applied to the neuromorphic system based on RRAM memristive synaptic devices (Pedretti et al., 2017). Based on a similar methodology, the detection of the coincidence of simultaneous spikes representing an image among noise was developed (Sebastian et al., 2017; Prezioso et al., 2018) (Figure 5A).

Spatiotemporal spiking patterns can also be learned in a memristive neuromorphic system via a modified STDP learning rule, where the potentiation and depression of memristive synapse can be related to the precise timing of its received spike (Wang et al., 2018b) (Figure 5B). This can potentially enable direct learning and recognition of spatiotemporal signals in the real world, such as speech, motion, and gesture recognition (Wang et al., 2019a).

Unsupervised learning based on STDP like learning rule has been demonstrated in a fully memristive neural network integrated with memristive synapse and diffusive memristor-based neurons. Pattern classification has been realized with such fully memristive neural network after unsupervised learning (Wang et al., 2018).

#### Collective-State Computing

Hopfield networks realistically describe neurophysiological processes and exhibit associative memory behaviors with the system automatically evolving to attractor states (Hopfield 1982). In the Hopfield network, each neuron receives input from all other neurons, and integrate-and-fire neurons can be employed (Eryilmaz et al., 2014). Thus, when a fixed spiking pattern is presented to the neurons, the synapse can receive overlapping stimuli between self-generated spikes and the input spikes in its two terminals (Figure 5C). The synapse weight can thus be updated with Hebbian-like rules, such as STDP (Milo et al., 2017). The learned configuration of synaptic weights forms an attractor state. After the learning, even if only part of the spiking pattern is presented to the neurons, the full spiking pattern can be recalled (Milo et al., 2018), which is the basic concept of associative memory or content-addressable memory. The number of attractors that can be learned in a single synaptic array largely depends on the size of the network and is also affected by the learning rules. The Oja rule is reported to have a larger memory capacity roughly 10 times better than the Hebbian rule (Wang et al., 2020a).

The cellular neural network only allows local connections between neighboring neural cells (Chua and Yang 1988). In a standard cellular neural network, the neuron cells are arranged in a 2D array and the synapses bridge each cell with its neighboring cells (Duan et al., 2015). Many two-dimensional tasks such as pattern and image analysis can be solved parallelly with such a 2D arrangement of cellular neural networks. Owing to the localized synaptic connections or communication between cells and the fully parallel operations of each cell, it is more suitable to be implemented with the hardware within the neuromorphic regime. Memristive synapses can further reduce the area cost compared with a CMOS only solution (Dominguez-Castro et al., 1997). Simulation results have shown that memristive cellular neural networks execute functions of image processing such as horizontal line detection, edge extraction, and noise removal (Duan et al., 2015). The non-linear I-V relation in memristive devices is incorporated in the analysis and simulation and has not proven to be an issue. However, the impact of other non-idealities of the memristive devices on the system performance needs further investigation. Disordered graphical network maps can be viewed as a special case of cellular neural networks. Theoretical analysis of these networks based on memristive connections shows much richer dynamic behaviors (Caravelli et al. 2017).

The oscillation network is another example of collective-state computation. Coupled with a memristive circuit, two self-sustained relaxation oscillators show frequency synchronization and phase locking (Ignatov et al., 2016). This is believed to convey two essential principles of biological computing, namely, synchronization and memory. More recently, the memristive-coupled oscillator network is extended for temporal binding of different attributes of the same object (Marina Ignatov et al., 2017). In another work, the oscillators are implemented by spin-torque memristive devices (Figure 5D), while the coupling factors among the oscillators are tuned by the direct current through each oscillator (Romera et al., 2018). Vowel recognition with four coupled spin-torque oscillators was experimentally demonstrated.

### Outlook

The integration of the memristor-based neuromorphic computing systems requires a detailed co-design at various levels, ranging from material optimization to system engineering. At each level, there are various integration methods depending on the approach and goal of the final system. Joint efforts and collaborations from experts in various research fields are needed. This perspective clarifies the goals of the efforts at various integration levels for two approaches to memristive neuromorphic systems: the DL accelerator and the brain-like computation.

The implementation of state-of-the-art DL techniques enabled by the material- and device-level optimization and by the array level adaption has been a fruitful exploration in memristive neuromorphic computing. It can be viewed as a model for the co-design of memristive devices and algorithms. This methodology mainly relies on the maturity of DL algorithms and the popularity of these techniques in the AI era. Thanks to this popularity, materials scientists and electrical engineers working on memristive devices have sufficient prior knowledge to explore current machine learning infrastructures and slightly modify the algorithms as needed for real situations encountered in memristive synapses. Prototype systems realizing benchmark cognitive functions, for instance, the image classification for MNIST dataset, CIFAR, have been demonstrated or simulated (Yao et al., 2020; Ambrogio et al., 2018). However, demonstrations of large-scale fully integrated memristive neuromorphic solutions for DL acceleration beyond the relatively small tasks (e.g., MNIST, CIFAR), toward more practical applications (e.g., in the scale of ImageNet [He et al., 2016]), are still lacking.

Exploiting unique features, including those traditionally viewed as non-idealities, of memristive devices enables a more direct and efficient implementation of brain-inspired algorithms, resulting in artificial synapses, dendrites, and neurons closely resembling their biological counterparts, as well as some basic functionalities in biological systems. However, these brain-inspired algorithms do not directly result in practical computational capabilities. Compared with memristive DL accelerators, memristive brain-like computations are limited to a smaller scale or toy applications so far. Besides the technical issues that need to be addressed, memristive SNNs are relatively underdeveloped mainly because of the lack of a clear understanding of biological information representations and processes that occur in the brain.

SNN and collective-state computation are two possible frameworks, both resembling essential features of biological computations, to utilize the brain-inspired algorithms for brain-like computation. The artificial synapses, neurons, and dendrites that can faithfully emulate their biological counterparts may eventually provide building blocks for bio-realistic artificial neural networks. Such neural networks not only serve as computation tools that can generate natural intelligence but also act as faithful biological emulators to verify neuroscience principles. Compared with the biological tissues that essentially compose a “Blackbox” for neuroscience experiments, such an electronic testbed could be considered a “Whitebox” where every node in the neural network can be monitored, measured, and understood. In this way, memristor-based brain-like neural networks will not only benefit from, but also be beneficial for, the understanding of how biological neural networks naturally process information. Co-design between memristive hardware and neural network algorithms is critical for developing such brain-like neural networks.
